# Serum levels of IgM to phosphatidylcholine predict the response of multiple sclerosis patients to natalizumab or IFN-β

**DOI:** 10.1038/s41598-022-16218-y

**Published:** 2022-08-03

**Authors:** Úrsula Muñoz, Cristina Sebal, Esther Escudero, Elena Urcelay, Rafael Arroyo, Maria A. García-Martínez, Francisco J. Quintana, Roberto Álvarez-Lafuente, Maria Cruz Sádaba

**Affiliations:** 1 Facultad de Medicina, Instituto de Medicina Molecular Aplicada (IMMA), Universidad San Pablo-CEU, CEU Universities, Crta Boadilla del Monte Km 5,3, Madrid, Spain; 2 Instituto de Investigación Sanitaria San Carlos (IdISSC)/Hospital Clínico San Carlos, Madrid, Spain; 3 Departamento de Neurología, Hospital Universitario Quironsalud Madrid, Madrid, Spain; 4 Grupo de Investigación de Factores Ambientales en Enfermedades Degenerativas, Instituto de Investigación Sanitaria San Carlos (IdISSC)/Hospital Clínico San Carlos, Madrid, Spain; 5 Ann Romney Center for Neurologic Diseases. Brigham and Women’s Hospital, Harvard Medical School. Associate Member, The Broad Institute, Boston, USA

**Keywords:** Biochemistry, Biological techniques, Biotechnology, Neuroscience, Immunosuppression, Demyelinating diseases, Multiple sclerosis, Predictive markers, Immunology, Autoimmunity

## Abstract

We developed an ELISA assay demonstrating the high prevalence of serum IgM to phosphatidylcholine (IgM-PC) in the first stages of multiple sclerosis (MS). We aimed to analyze the role of serum IgM-PC as a biomarker of response to treatment. Paired serum samples from 95 MS patients were obtained before (b.t) and after (a.t) treatment with disease modifying therapies. Patients were classified as non-responders or responders to treatment, according to classical criteria. Serum IgM-PC concentration was analyzed using our house ELISA assay. The level of serum IgM-PC b.t was higher in patients treated later with natalizumab than in those treated with Copaxone (*p* = 0.011) or interferon-β (*p* = 0.009). Responders to natalizumab showed higher concentration of serum IgM-PC b.t than those who did not respond to it (*p* = 0.019). The 73.3% of patients with the highest level of serum IgM-PC b.t responded to natalizumab. IgM-PC level decreased a.t in both cases, non-responders and responders to natalizumab. IgM-PC levels a.t did not decrease in non-responders to interferon-β, but in responders to it the IgM-PC level decreased (*p* = 0.007). Serum IgM-PC could be a biomarker of response to natalizumab or interferon-β treatment. Further studies would be necessary to validate these results.

## Introduction

Multiple sclerosis (MS) is a demyelinating and neurodegenerative disease of the central nervous system (CNS). Lipids are the main component of myelin, and antibodies to sulfatide, ganglioside GM4, galactocerebroside and cholesterol^1^ have been detected in the cerebrospinal fluid of MS patients. Moreover, IgM-PC in cerebrospinal fluid is associated with a more aggressive disease form of MS, characterized by more frequent relapses, faster progression of the EDSS, and rapid evolution to the progressive phase of the disease^2^. Recently, we detected IgM on axons and oligodendrocytes in MS brain samples, co-localizing with activation complement factors^3^. Moreover IgM co-localizes with markers of cellular and axonal damage^4^.

The analysis of the peripheral compartment has also demonstrated the presence of antibodies to lipids in MS. Serum IgG antibodies to lactosylceramide are associated with cerebral tissue damage in these patients^5^. Recently, we developed a sensitive assay, which detected serum IgM-PC in almost 90% of MS patients during the initial phases of the disease^1^. These and additional data suggest a role of antibodies as potential biomarkers in MS.

Treatments delay MS progression, but do not cure it. In addition, 40% patients do not respond to first-line treatments^6,7^, and most available treatments show limited efficacy in the progressive phase of disease^8^. Moreover, side effects, such as cardiomyopathy, leukemia, progressive multifocal leukoencephalopathy, bradyarrhythmia, macular edema, herpes zoster virus infections, autoimmune thyroiditis, thrombocytopenia and glomerulonephritis are associated to immunosuppressive therapies^9^. Thus, it is important to characterize biomarkers of treatment response to develop personalized therapies, improving their effectiveness while minimizing their side effects. With this goal in mind, we evaluated the role of serum IgM-PC as a biomarker of response to MS treatment.

## Methods

### Classification criteria

This is a Class II criteria study with retrospective sample and clinical data collection from MS patients^10^. The analytical assay (ELISA to quantify the IgM-PC levels) and the clinical data collection were developed by different researchers in a double-blind study.

### Patients

A total of 95 MS patients were included in this study. All the protocols were approved by the Bioethics Committee of Hospital Clínico San Carlos (Madrid, Spain) and Committee of Bioethics of Hospital Universitario Quirónsalud (Madrid, Spain).

All the patients gave verbal and written informed consent for sample collection.

All methods were carried out in accordance with relevant guidelines and regulations (Real Decreto 1716/2011 de 18 de noviembre, Government of Spain)^11^.

Paired samples were obtained before treatment and 6 months after starting it, and were aliquoted and stored at -80º C until analyzed.

Patients were classified depending on the response to treatment^12^, considering the progression of the disease and the number of relapses. To assert progression, we considered the following criteria: (1) increase ≥ 1.5 points at 24-months visit, if pre-treatment EDSS = 0; (2) increase ≥ 1 point at 24-months visit, if pre-treatment EDSS was ≥ 1 and ≤ 5; (3) increase ≥ 0.5 points at 24-months visit, if pre-treatment EDSS was ≥ 5.5. Relapses were uttered as a worsening of neurological impairment or a new neurologic dysfunction affecting a different area of the central nervous system, with a duration of more than 24 h and preceded by stability of at least 1 month. Based on these criteria, we stated the clinical response to treatment (defined as an absence of relapses and disability progression) and the therapeutic failure (≥ 2 relapses and/or disability progression) after two years of follow-up.

The demographic and clinical data, treatment, and response of all patients analyzed are summarized in Table 1.

**Table 1:** Demographic and clinical data from MS patients.

Treatment	Response	Sex (Females)	Age (mean ± SD)	EDSS increase in two years (mean ± SD)	Relapses after treatment (mean ± SD)
Natalizumab	NO (*n* = 18)	50%	40.5 ± 2.43	0.64 ± 0.21	1.22 ± 0.31
	YES (*n* = 16)	50%	34.67 ± 2.56	0.53 ± 0.21	0 ± 0
Copaxone	NO (*n* = 15)	46.70%	40.83 ± 2.75	0.63 ± 0.19	1.13 ± 0.19
	YES (*n* = 12)	33.30%	35 ± 2.78	0.25 ± 0.2	0 ± 0
Avonex	NO (*n* = 6)	50.00%	46 ± 0	1.25 ± 0.6	0.83 ± 0.4
	YES (*n* = 4)	75.00%	36 ± 1	0.88 ± 0.38	0 ± 0
Rebif	NO (*n* = 7)	57.10%	28 ± 7.5	0.28 ± 0.42	1.57 ± 0.43
	YES (*n* = 6)	33.30%	25 ± 0	0.33 ± 0.31	0 ± 0
Betaferon	NO (*n* = 4)	50.00%	36.5 ± 7.5	0.5 ± 0.71	1.25 ± 0.63
	YES (*n* = 7)	71.40%	35 ± 5.74	0.64 ± 0.32	0 ± 0

*NO* patients who do not respond to treatment, *Yes* patients respond to treatment, *SD* standard deviation.

### ELISA assay

To detect IgM antibodies to PC, we used a method we published previously^1^. Briefly, 96-well plates were coated with PC (Sigma-Aldrich, St. Louis, MO). After washing three times with phosphate-buffered saline, the wells were blocked. Serum samples were diluted (1/50) in blocking solution and pipetted by triplicate into the wells. IgM antibodies were detected with anti-human IgM biotin (Jackson ImmunoResearch, West Grove, PA), followed by streptavidin–horse peroxidase (Sigma- Aldrich). Finally, we used TMB-one (Thermofisher Scientific Inc) as substrate. Plates were read at 450 nm using a Varioscan Flash spectrophotometer (Thermofisher Scientific). The level of antibodies was described as optic density (O.D). To determine variations in the level of IgM-PC before (b.t) and after (a.t) the treatment, we used this equation: ((O.Da.t-O.Db.t)/O.Db.t) × 100. We asserted variations when the value was ± 5%.

### Statistics

Statistical analyses were performed with GraphPad Prism (version 6.0) and IBM SPSS 24 statistical packages; *p* values < 0.05 were considered statistically significant. Mann Whitney test was used to compare IgM-PC levels in patients treated with different drugs, and in non-responders and responders. We used the Wilcoxon test to compare the IgM-PC concentration in serum samples before and after treatment. To analyze the percentage of non-responders and responders in the quartiles of IgM-PC concentration or the percentage of patients showing decrease, no changes or increase in IgM-PC levels after the treatment we used the χ^2^ test.

## Results

### High levels of IgM-PC.b.t predict the response to natalizumab

To assert the role of IgM-PC as a biomarker of response to treatment, we analyzed the IgM-PC levels in serum samples obtained before the administration of the different disease modifying therapies (IgM-PC.b.t) by using the highly sensitive technique developed in our laboratory^1^.

IgM-PC.b.t was neither related to sex, age, nor disease duration.

**Figure 1:**
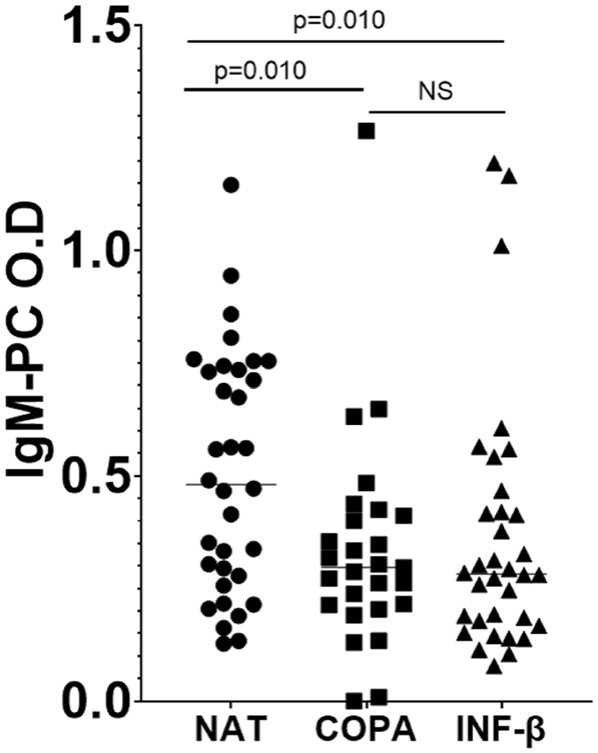
IgM-PC.b.t concentration measured as O.D in serum samples from MS patients. Dots, squares, and triangles represent the concentration obtained in every individual. The transverse line represents the median. *IgM-PC.b.t* Concentration of serum IgM to PC before treatment. *IgM-PC O.D* Concentration of IgM to PC before treatment measured as optic density (O.D). *NAT* Natalizumab. *COPA* Copaxone. *INF-β* interferon-β.

We could observe that the levels of IgM-PC.b.t were higher in patients treated with natalizumab (0.481 ± 0.045, median ± standard deviation) than in those treated with Copaxone (0.297 ± 0.046; *p* = 0.010) or IFN-β (interferon-β) (0.282 ± 0.048; *p* = 0.010). No significant differences were detected between patients treated with Copaxone or interferon-β (Fig. 1).

Then, we studied the relation between the IgM-PC.b.t levels and the response to treatment.

**Figure 2:**
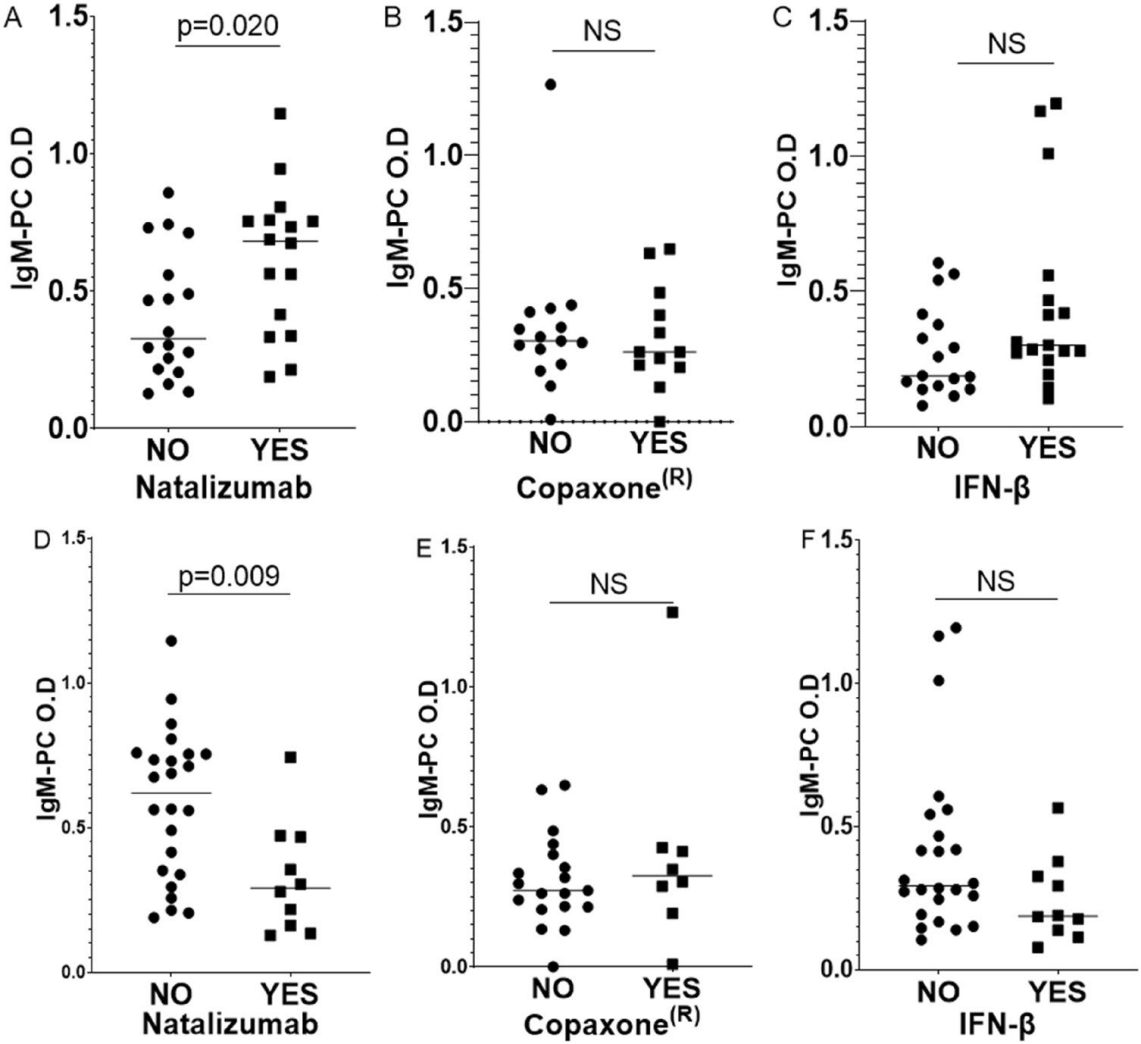
Analysis of IgM-PC.b.t levels in serum samples from MS patients. IgM-PC.b.t in patients treated with natalizumab (**A**), (**D**), Copaxone (**B**), (**E**), and interferon-β (**C**), (**F**). (**A**–**C**) Relation between the IgM-PC levels and the treatment response. (**D**–**F**) Relation between the IgM-PC levels and treatment failure. Dots and squares represent the IgM-PC concentration obtained in every individual. The cross line represents the median. *IgM-PC.b.t* Concentration of serum IgM to PC before treatment. *O.D* Concentration of IgM-PCb.t measured as optic density (O.D). (**A**–**C**) *NO* non-responders. *YES* responders. (**D**–**F**) *NO* non treatment failure. *YES* treatment failure.

We did not detect a relationship between the IgM-PC.b.t levels and the response to treatment when Copaxone or IFN-β where administrated. However, responders to natalizumab had a higher IgM-PC.b.t level (0.618 ± 0.065) compared with those who did not respond (0.327 ± 0.054; *p* = 0.020) (Fig. 2A–C).

Similar results were observed when the relation between IgM-PC.b.t and treatment failure was analyzed. No association between IgM-PC.b.t levels and therapeutic failure was detected when Copaxone or interferon-β were administrated. Conversely, patients who did not suffered therapeutic failure with natalizumab showed a higher IgM-PC.b.t concentration (0.583 ± 0.0.52) than those who did (0.397 ± 0.044; p = 0.009) (Fig. 2D–F).

**Figure 3:**
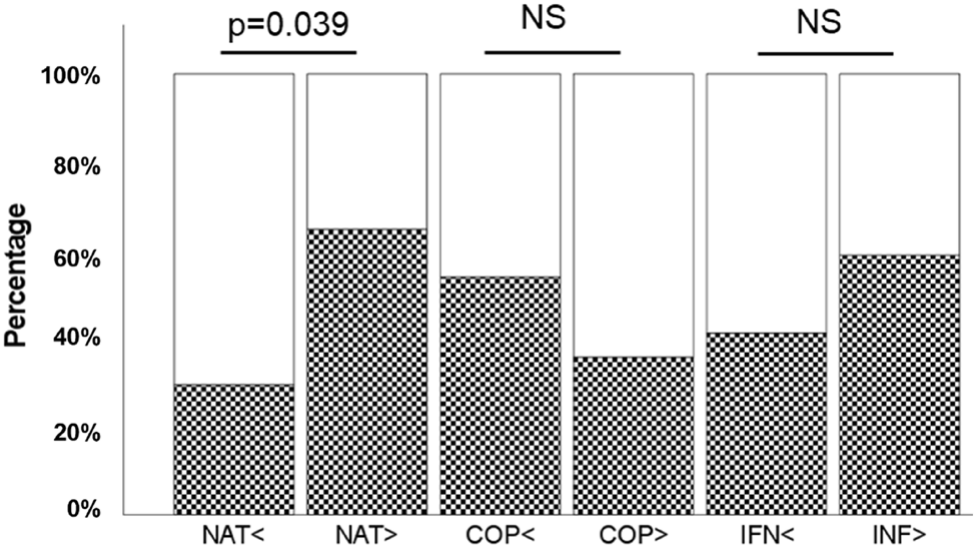
Relation between the IgM-PC.b.t levels and the response to disease modifying therapy. White bars: percentage of non-responders. Squared bars: percentage of responders. *NAT* Natalizumab. *COP* Copaxone. *IFN* interferon-β. < Patients with IgM-PC.b.t level below the median. > Patients with IgM-PC.b.t level above the median.

To assess the role as a prognosis marker of response to the different treatments, we analyzed the probability of response or not response to the treatment in patients with IgM-PCb.t levels above or below the median. We could observe that 68.8% of patients with IgM-PC.b.t levels above the median responded to natalizumab, compared with 31.3% of those who did not. However, the probability of response of patients treated with Copaxone or interferon-β was independent of the IgM-PC levels (Fig. 3).

To determine the IgM-PC O.D cutoff that predicted the response to natalizumab with complete certainty, we classified the IgM-PC.b.t levels in quartiles from Q1 (lower IgM-PC.b.t levels) to Q4 (higher IgM-PC.b.t levels) and then, we analyzed the probability of responding to treatment for each one.

**Figure 4:**
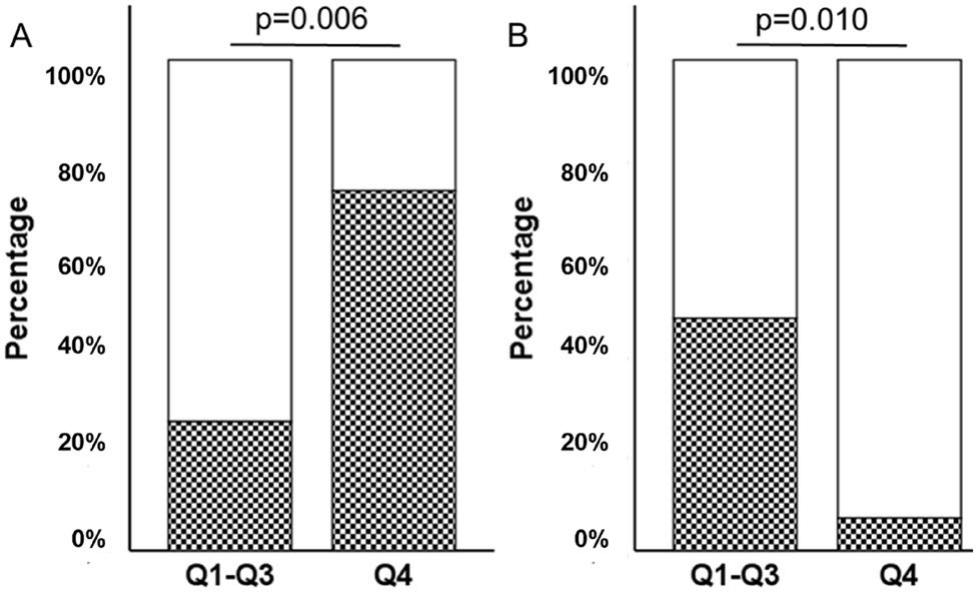
Relation between the levels of IgM-PC.b.t determined as quartiles, and the response to treatment or the treatment failure when natalizumab was used. (**A**) White bars: percentage of non-responders. Squared bars: percentage of responders. (**B**) White bars: percentage of non-treatment failure. Squared bars: percentage of treatment failure. The levels of serum IgM-PCb.t were classified as quartiles. *Q1–Q3* Low-medium IgM-PCb.t levels. *Q4* high gM-PCb.t levels.

We observed that 73.3% (11/15) of patients in Q4 responded to natalizumab, but only 26.3% (5/19) in Q1-Q3 responded to this drug (*p* = 0.006) (Fig. 4A). In contrast, no significant differences between quartiles were detected when patients treated with Copaxone or interferon-β were analyzed (data not shown).

The analysis of IgM-PC.b.t levels related to treatment failure demonstrated similar results: 93% of patients treated with natalizumab in Q4 did not suffer a treatment failure, compared with 52.6% of patients in Q1-Q3 (*p* = 0.010) (Fig. 4B). However, the classification of IgM-PC.b.t levels in quartiles did not discriminate the response to Copaxone nor interferon-β (data not shown).

### Effect of treatment on serum IgM-PC levels in MS patients.

To study in detail the role of IgM-PC as a prognosis biomarker, we analyzed the effect of the different treatments on the level of these immunoglobins, comparing the levels of IgM-PC before and after the treatment (IgMPC.a.t) in paired samples from MS patients (Table 2).

**Table 2:** IgM-PC level in serum paired samples before and after the treatment and the difference between them.

Treatment	Response	IgM-PC (O.D) B.T	IgM-PC (O.D) A.T	Difference A.T–B.T	Variation % A.T–B.T
Natalizumab	NO (*n* = 18)	0.409 ± 0.054	0.269 ± 0.044	− 0.140 ± 0.031	− 35.14% ± 7.00
	YES (*n* = 16)	0.619 ± 0.066	0.414 ± 0.055	− 0.228 ± 0.050	− 40.29% ± 7.29
Copaxone	NO (*n* = 15)	0.351 ± 0.072	0.375 ± 0.077	0.023 ± 0.036	13.45% ± 17.00
	YES (*n* = 12)	0.318 ± 0.056	0.372 ± 0.070	0.055 ± 0.079	55.00% ± 40.90
Avonex	NO (*n* = 6)	0.287 ± 0.062	0.292 ± 0.069	0.005 ± 0.030	3.27% ± 12.94
	YES (*n* = 4)	0.256 ± 0.058	0.226 ± 0.080	− 0.030 ± 0.052	− 13.24% ± 22.61
Rebif	NO (*n* = 7)	0.260 ± 0.064	0.301 ± 0.051	0.040 ± 0.041	43.87% ± 35.66
	YES (*n* = 6)	0.277 ± 0.047	0.218 ± 0.049	− 0.058 ± 0.024	− 27.88% ± 14.79
Betaferon	NO (*n* = 4)	0.296 ± 0.113	0.366 ± 0.172	0.069 ± 0.068	19.13 ± 21.04
	YES (*n* = 7)	0.709 ± 0.151	0.426 ± 0.119	− 0.283 ± 0.153	− 33.20 ± 13.84

*O.D* optic density, *B.T* before treatment, *A.T* after treatment, *NO* non responders, *YES* responders. Data represent mean ± standard deviation.

**Figure 5:**
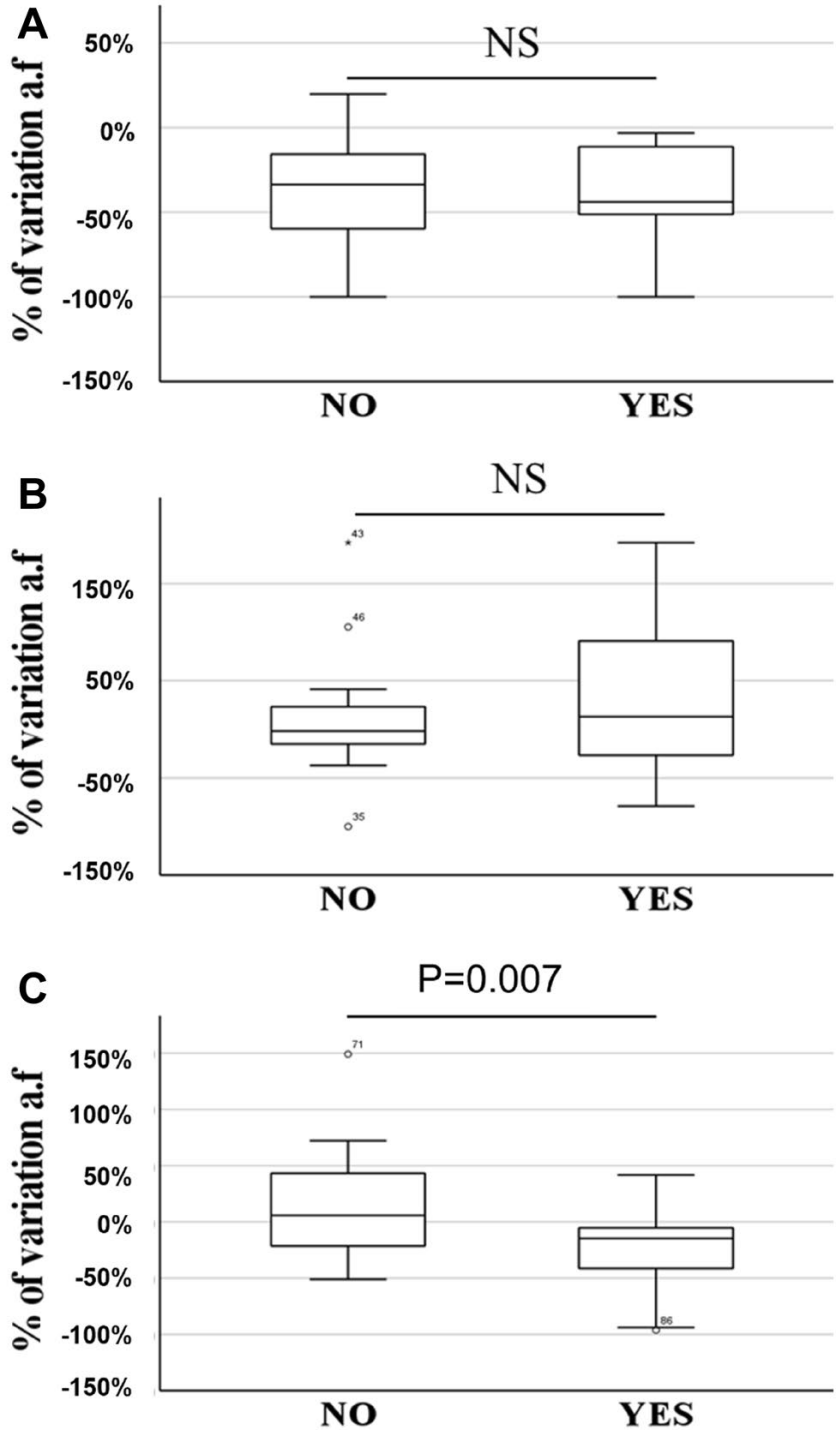
Study of the variations of IgM-PC levels after treatment in serum samples from no responders (NO) and responders (YES) MS patients to treatment with natalizumab (**A**), Copaxone (**B**) and interferon-β. Boxes represent the median of relative decrease ± percentiles 25–75, and whiskers include 100% of the patients.

We observed a similar decrease of IgM-PC concentration after the treatment with natalizumab in both groups, non-responders (− 36.14% ± 7.00%) and responders (− 40.29% ± 7.29%) (Fig. 5A). On the contrary, the serum IgM-PC levels did not decrease after the treatment with Copaxone, neither non-responders (13.45% ± 17.00%), nor responders (55.00% ± 40.90%) (Fig. 5B). The levels of IgM-PC.a.t did not decrease in non-responders (23.72% ± 15.95%) to interferon-β, but they decreased significantly (*p* = 0.007) in responders to this drug (− 26.63 ± 8.95) (Fig. 5C).

To assess the role of IgM-PC levels as a prognosis biomarker, we aimed to determine the probability of response to treatment in those patients showing decreased IgM-PC levels. We established three groups of patients: without variation in serum IgM-PC antibody levels, and with increased or decreased serum IgM-PC levels, in clinical responders and non-responders.

**Figure 6:**
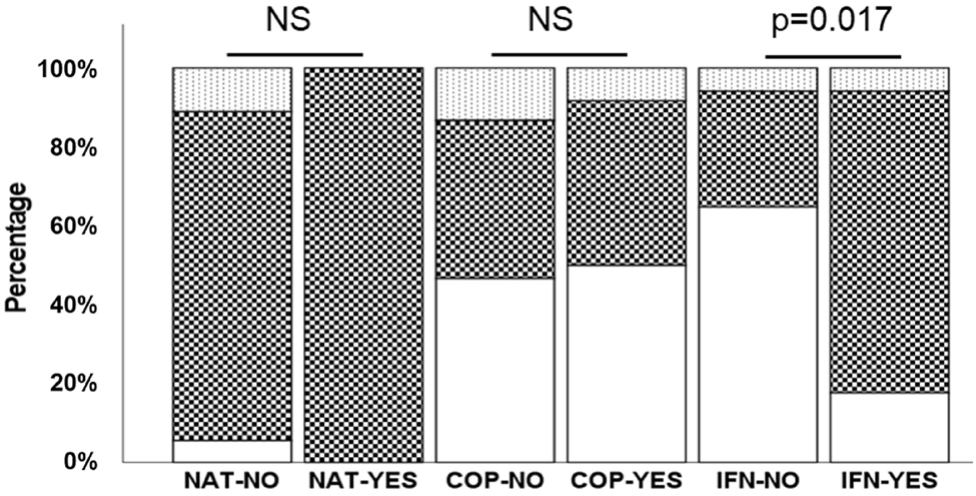
Analysis of the percentage of patients showing decrease, non-change or increase in IgM-PC levels after treatment. White bars: non-changes in IgM-PC levels. *Squared bars* decreased IgM levels. *Dotted bars* increased IgM-PC levels. *NAT* Natalizumab. *COP* Copaxone. *IFN* interferon-β. *NO* nonresponse to treatment. *YES* response to treatment.

Natalizumab treatment led to a decrease in serum IgM-PC in most patients (91.18%); we did not detect differences between responders and non-responders. In patients treated with Copaxone, we did not find differences in IgM-PC variation in clinical responders nor in non-responders. In contrast, 76.5% of responders to interferon-β showed a decreased serum IgM-PC and only 29.4% of non-responders had a diminution of these immunoglobulins (*p* = 0.017) (Fig. 6).

## Discussion

Recently, our group has developed an ultrasensitive technique to detect serum IgM to PC. We demonstrated that almost 90% of the patients had IgM-PC in serum at the onset of the disease^1^, being the most sensitive and standardizable technique for the early diagnosis of MS^1^. The presence of antibodies to lipids in serum are related with tissue damage^5^, and the detection of oligoclonal IgM bands to lipids in the cerebrospinal fluid is a prognosis marker of poor evolution in MS^2^. In addition, we observed IgM co-localizing with markers of oligodendrocyte and axonal damage^4^ in brain samples from MS patients. These immunoglobulins can trigger the lysis of the cells, because they induce the activation of the complement cascade^3^. Regarding this, IgM is pentameric and activates the complement 1000 times more efficiently than IgG^13^.

IgM to lipids, DNA and carbohydrates form part of the termed “natural antibodies”^14^, the first line of defense to pathogens. As natural antibodies recognize phylogenetically conserved structures, they raise the elimination of a broad variety of microorganism, virus, bacteria, fungi, and parasites. The structure of IgM promotes the neutralization and agglutination of the microorganisms, preventing their dissemination. These antibodies trigger the pathogen destruction, activating the complement cascade and promoting phagocytosis, which in turn boasts the adaptive immune response^13^.

Natural antibodies can also bind to autoantigens, such as oxidized-LDL present in atherosclerotic plaques^13^, and phospholipids expressed by apoptotic cells. It is supposed that IgM facilitates the clearance of the latter, avoiding inflammatory processes^14^. Regarding this, the most accepted hypothesis in autoimmunity claims that the exposition to a microorganism would trigger the formation of autoantibodies capable to recognize both, microbial and self-antigens^15^.

The presence of IgM-PC indicates the elevated activity of B1 cells, marginal zone, or peritoneal B lymphocytes. These lymphocytes are only stimulated by their specific antigen and always produce IgM.

The efficiency of therapies focused on B lymphocytes also demonstrate the relevance of these cells in the physiopathology of MS^16^. Treatments delay the disease progression, but have significant side effects^9^. In addition, the response to treatment is very variable, and 40% of the cases do not respond to treatment and a change to another therapy is required^6^. Thus, numerous studies aimed to characterize biomarkers of response to treatment. This is essential to make clinical decisions, when to start the treatment or change to another drug. It is also important for the future, to develop more personalized therapies and improve patients’ response.

Considering these data, we hypothesized that disease modifying therapies could modulate the production of IgM to lipids, and we aimed to study the relation between the IgM-PC levels, detected using our highly sensitive ELISA, and the response to different treatments.

We could observe that patients treated with natalizumab showed higher basal IgM-PC levels than patients receiving Copaxone or interferon-β. This treatment is indicated when first-line drugs are not efficient and patients have a high number of relapses and a rapid EDSS progression^17^, reflection of a high inflammatory activity^18^. Moreover, most patients with a high IgM-PC concentration (those in the fourth quartile) responded to natalizumab. In this line, previous results indicate that the decrease of IgM in the cerebrospinal fluid is a biomarker of response to natalizumab^19^. Our results draw attention to the role of serum IgM-PC level as a biomarker of response to natalizumab.

In addition, most patients treated with natalizumab showed a significant decrease in the levels of IgM-PC despite of they responded or not to the treatment. These results are consistent with those demonstrating reduced levels of both, IgG and IgM in serum^20^ after treatment with this humanized antibody, because 80% of the circulating IgM consists of “natural antibodies” synthetized by B1 lymphocytes^14^. VLA-4 mediates the homing of B lymphocytes to peripheral lymph nodes^21^, splenic white pulp^22^, mesenteric lymph nodes, and Peyer’s patches^22^. Therefore, the administration of natalizumab increases the number of B cells in the blood more than other cells^23^. Different studies reported that the subpopulations affected are pre-B^23^, memory and marginal zone-like cells^24^. Unfortunately, there are not experimental evidences describing the effect of natalizumab on B1 lymphocytes. We hypothesize that anti-VLA inhibits the activation of B1 lymphocytes^25^, or their homing to lymphoid organs, where they have their niches and differentiate to plasmablasts^26^, main effectors in MS^27^.

It seems contradictory that different evidences support the role of IgM to lipids as a main pathological mechanism, but patients who did not respond to natalizumab showed reduced serum levels of these antibodies after treatment. This is the reflection of the existence of two different compartments, CNS and peripheral system^1^, and the characteristics of patients treated with this monoclonal antibody. Natalizumab inhibits mainly the egression of CD4 + cells and B cells^19^ from blood to the CNS^28^. In addition, natalizumab reduces the intrathecal synthesis of IgG and IgM, but do not eliminate the presence of oligoclonal IgG or IgM bands completely^19^. We observed plasma cells and IgM deposits^4^ in chronic demyelinating lesions from MS patients with a long evolution. Moreover, it was detected lymphoid follicle-like structures in the meninges, associated with the damage of the nervous system^29^. Therefore, natalizumab can not avoid the presence of resident B and plasma cells producers of antibodies against self-antigens of the central nervous system. In this sense, depletion of B-lymphocytes in these patients does not eliminate the presence of oligoclonal bands in the CSF^30^.

As described above, this is a second-line treatment, administered in those cases in which first-line drugs have failed^17^ in patients with a poor evolution. The main mechanism of action of first-line drugs, such as Copaxone or interferons, is to inhibit the activity of T-lymphocytes^31^. These evidences support that in these cases immunosuppressive therapies do not regulate this population, which also plays a main role in the disease^32^. Moreover, it was suggested that intrinsic neurodegenerative mechanisms could be involved, especially in patients in the progressive phase^33^, explaining the inefficiency of immunosuppressive therapies in these individuals^8^.

IgM-PC.b.t levels did not predict the response to Copaxone. Interestingly, this therapy did not drop the IgM-PC levels, neither in non-responders nor in responders. These were expected results, because its main mechanism of action is to inhibit autoreactive T lymphocytes. This copolymer binds to many MHC molecules, inhibiting the response to different antigens^34^, and prevents the response of T lymphocytes to MBP, a major protein of the myelin. The inhibition of CD4 T lymphocytes could explain the suppression of experimental allergic encephalomyelitis by this polymer^34,35^. Patients treated with this drug showed increased levels of IL-10, IL-4 in serum, an anti-inflammatory profile^35,36^ that promotes the skewing towards TH2 responses. Regarding these, Copaxone reduces the number total number of B cells, plasmablast and memory B cells^37,38^. Probably, the affected subpopulation are the B2-lymphocytes, because B cells obtained from patients treated with the copolymer did not proliferate in response to CD40L^39^, the pathway used by T lymphocytes to activate this B2 subpopulation. However, B1 lymphocytes, the minority B subset in blood, are T independent^14^.

The levels of IgM-PC did not decrease in patients who did not respond to interferon-β. However, the immunoglobulin concentration dropped in patients who responded to this treatment. A decrease in these antibodies was observed in almost 80% of the responding patients. These data demonstrate that the study of IgM-PC could be a biomarker of response to treatment with interferon-β. It was described the relation between the concentration of different inflammatory molecules, such as, IL-17A^40^, IFN-ɣ, TNF-α^41^ IL-2^42^, TRAIL^41^ and CXCL13^40^, and the response to interferon-β. Another possible biomarker of response to treatment is the quantification of neurofilament heavy and light chains, a marker of axonal damage^40^. Nevertheless, they are not used in daily clinical practice. On the other hand, the detection of IgM-PC as a biomarker of prognosis has large advantages, the technique is cheap and standardizable, the interpretation of results is easy, and serum samples are used. In summary, this assay could be used routinely in most clinical laboratories. Moreover, the diminution of IgM-PC concentration in patients who do not experience relapses or increased disability after treatment could demonstrate that these immunoglobulins are a major pathologic mechanism in MS. These data are in line with other previously published, since interferon-β decreases the number of memory and activated B cells^43^. This could be mediated by the activation of the FAS-FASL pathway, inducing apoptosis of B cells^44^. However, the physiology and regulation of B lymphocytes producers of antibodies to lipids, and the effect of the disease modifying therapies in this subpopulation remains unknown. Our results support that interferon-β regulates this subpopulation. This could be of great relevance to characterize new therapeutic targets and also for the basic knowledge of the functioning of the immune system. In the future, it would be of great interest to study the functioning of these cells in patients who respond and do not respond to the treatment, in order to offer more personalized therapies and obtain greater effectiveness in the disease treatment.

In summary, these data could have a significant impact at the clinical level, but further studies are necessary to validate these results in larger cohorts of patients.

## Data Availability

The datasets generated during and/or analyzed during the current study are available from the corresponding author on reasonable request.
